# Supplementary material for: Connectivity between long-term care homes and subsequent SARS-CoV-2 outbreaks
Source: BMC Public Health. 2025 Aug 2;25:2634. doi: 10.1186/s12889-025-23621-3 (PMC12317546; doi:10.1186/s12889-025-23621-3)
Supplement: Supplementary file 2 — Supplementary Material 2. [file 12889_2025_23621_MOESM2_ESM.docx]

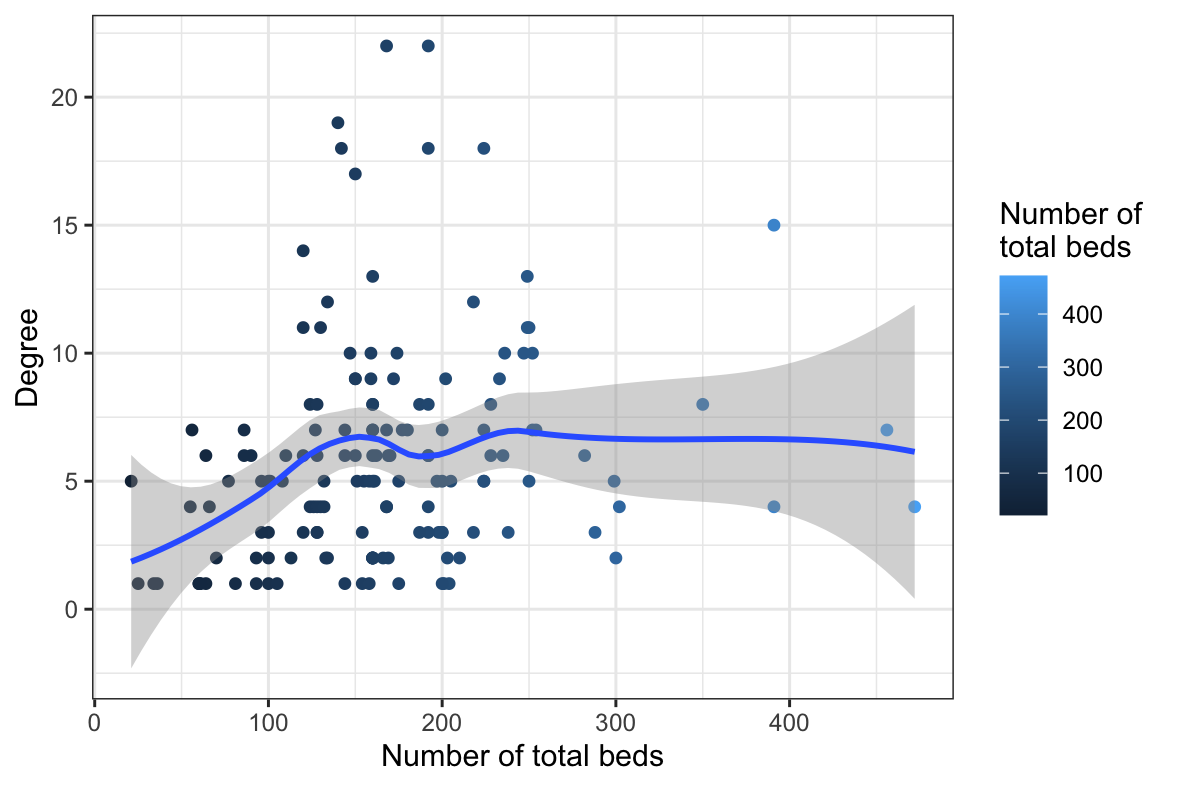


**Figure S1**. Degree of connectivity of long-term care homes in Greater Toronto Area, Ontario by number of total beds.


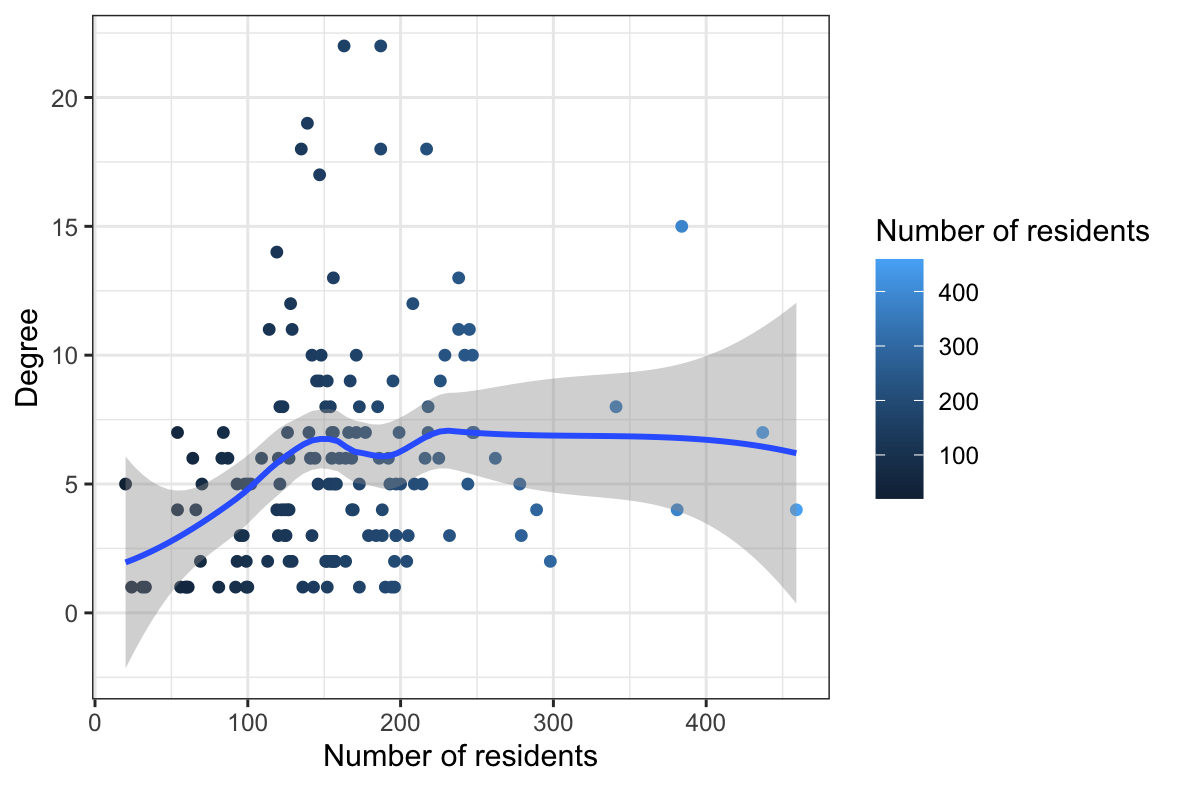


**Figure S2**. Degree of connectivity of long-term care homes in Greater Toronto Area, Ontario by number of residents.


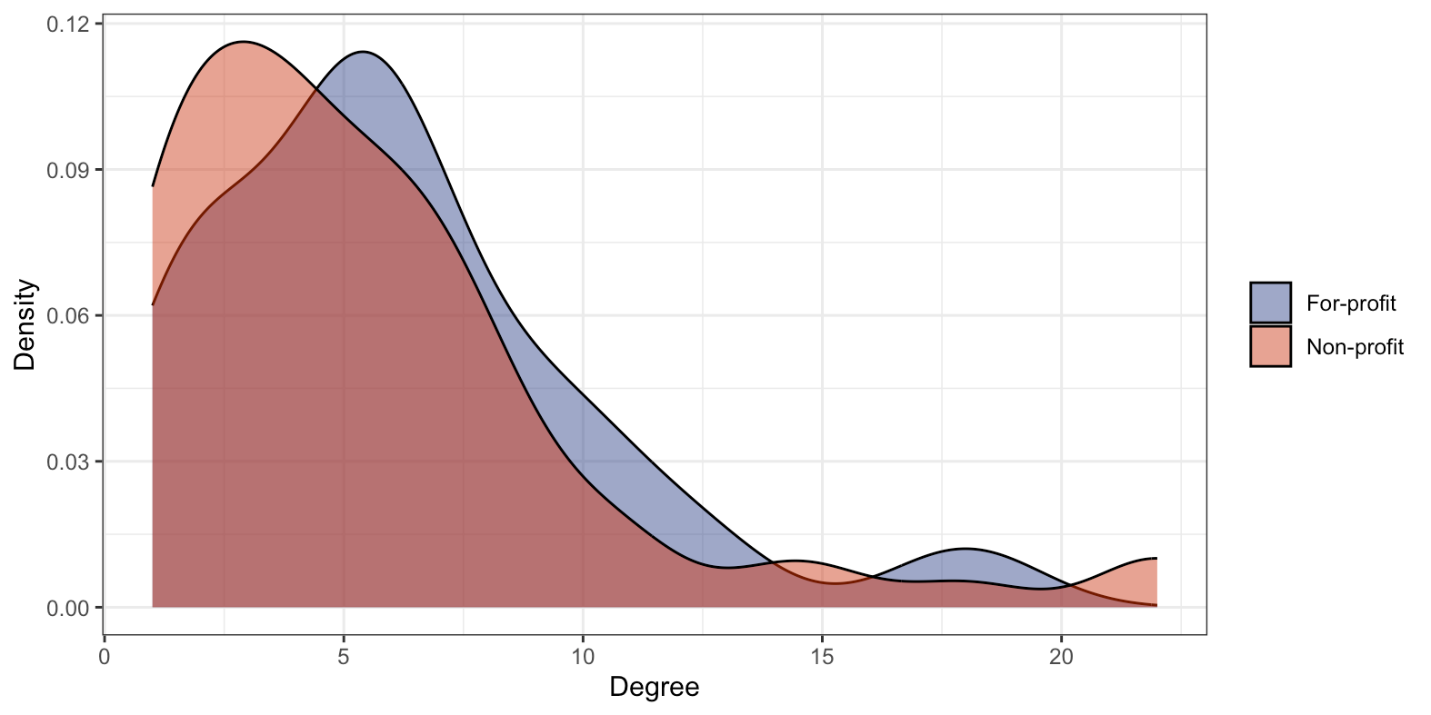


**Figure S3**. Degree of connectivity of long-term care homes in Greater Toronto Area, Ontario by for-profit status.


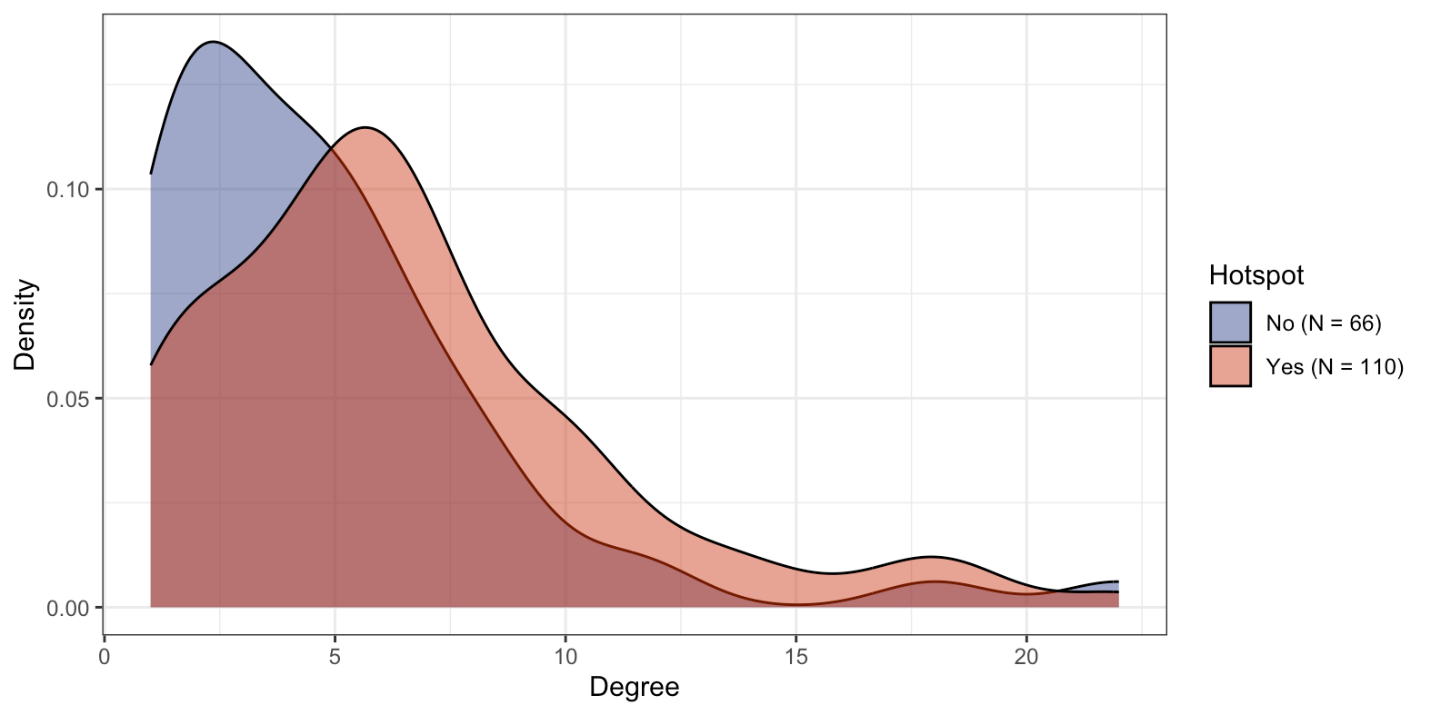
**Figure S4**. Degree of connectivity of long-term care homes in Greater Toronto Area, Ontario by hotspot status.


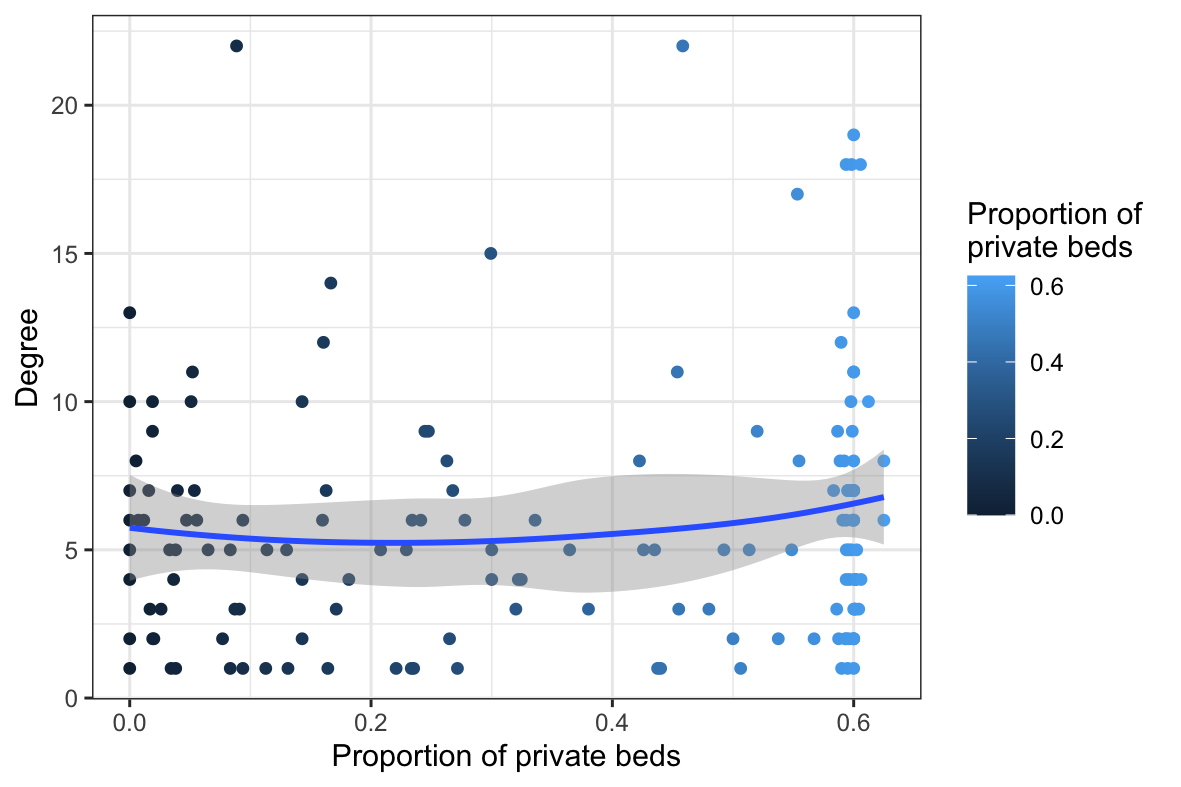


**Figure S5**. Degree of connectivity of long-term care homes in Greater Toronto Area, Ontario by proportion of private beds.
